# Supplementary material for: HCK induces macrophage activation to promote renal inflammation and fibrosis via suppression of autophagy
Source: Nat Commun. 2023 Jul 18;14:4297. doi: 10.1038/s41467-023-40086-3 (PMC10354075; doi:10.1038/s41467-023-40086-3)
Supplement: Supplementary file 1 — Supplementary Information [file 41467_2023_40086_MOESM1_ESM.pdf]

Supplementary Material for
HCK induces macrophage activation to promote renal inflammation and fibrosis via suppression of autophagy

This PDF file includes:
Supplementary Figures 1 to 5
Supplementary Table 1

Supplementary Figure S1: HCK is highly enriched in macrophages and the expression level upregulated in diseased kidneys.

Supplementary Figure S2. HCK KO in BMDMs impacted cell spreading by integrin signaling.

Supplementary Figure S3. High dosage of dasatinib caused proteinuria in lupus mice.

Supplementary Figure S4. Chemical strictures of 33 designed and synthesized compounds HCK inhibitors.

Supplementary Figure S5. Synthesis procedure of 3-(6,8-dichloro-2-phenyl-2H-chromen-3-yl)-5-phenyl-4,5-dihydro-1,2,4,5-oxadiazaborole (BT-424).

Supplementary Table S1. Serum chemistry test for mice treated with HCK selected inhibitor BT424 and vehicle.

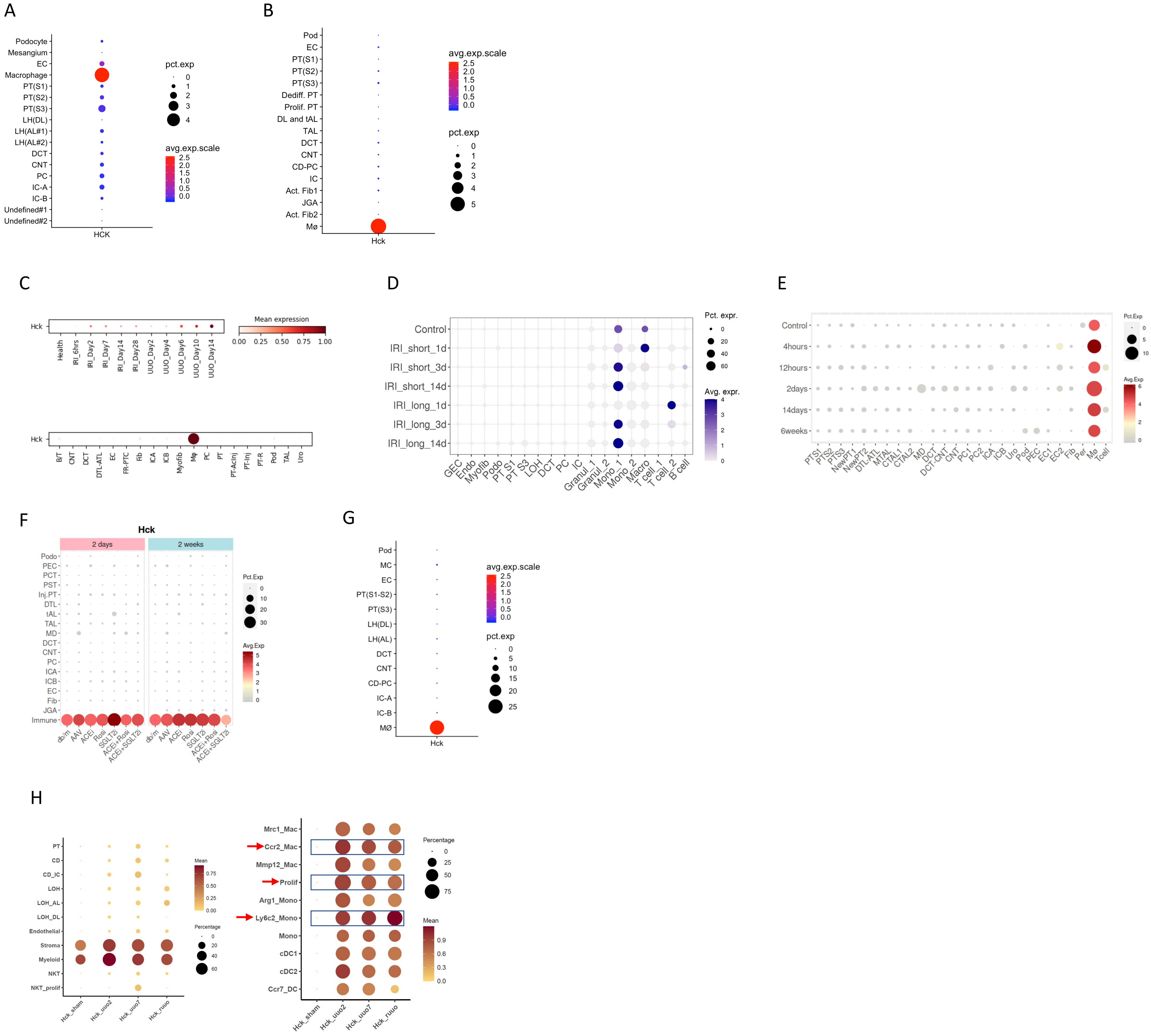

Figure S1: HCK is highly enriched in macrophages and the expression level upregulated in diseased kidneys.

(A, B) HCK is expressed in macrophages at low level in health kidneys. (A) Healthy Adult Kidney: Wu and Uchimura et al, Cell Stem Cell 2018. (B) Healthy Mouse Dataset: Wu et al, JASN 2019.
(C-E) HCK's expression level upregulated in diseased kidneys, comparing to health control kidneys. (C) Mouse IRI and UUO (Sham, IRI 6h 2d 7d 14d 28d, UUO 2d 7d 14d). Haikuo Li et al: In press. (D) Mouse IRI Kidney (short and long IRI, sham, 1d, 3d, 14d): Susztak etal, Nature Comm 2022. (E) Mouse IRI Kidney (Sham, 4hrs, 12hrs, 2days, 14days, and 6weeks): Kirita et al, PNAS 2020.
(F, G) HCK is expressed highly in macrophages in diseased kidneys. (F) 1 million cell atlas of mouse DKD and its treatments: Wu et al, Cell Metab 2022. (G) Mouse UUO (d14): Wu et al, JASN 2019. **(H)** HCK is mainly expressed in inflammatory (CCR2), infiltrating (Ly6) and proliferating (Mki67) macrophages subpopulation in diseased kidneys. (H) Mouse UUO (sham, 2d, 7d, R-UUO): Conway et al, JASN 2020.
EC, endothelial cells; PT, proximal tubular cells; LH, loop of Henle cells; DCT, distal convoluted cells; CNT, connecting tubular cells; PC, principal cells; IC-A, intercalate cells type A (located in the collection duct at the distal nephron); IC-B, intercalate cells type B (located in the collection duct at the distal nephron). Pod, podocyte; DL, descending loop; tAL, thin ascending loop; TAL, thick ascending loop; CD-PC, collecting duct – principal cell; IC, intercalated cell; Act. Fib., activated fibroblasts; JGA, juxtaglomerular apparatus; Mϕ, macrophage.

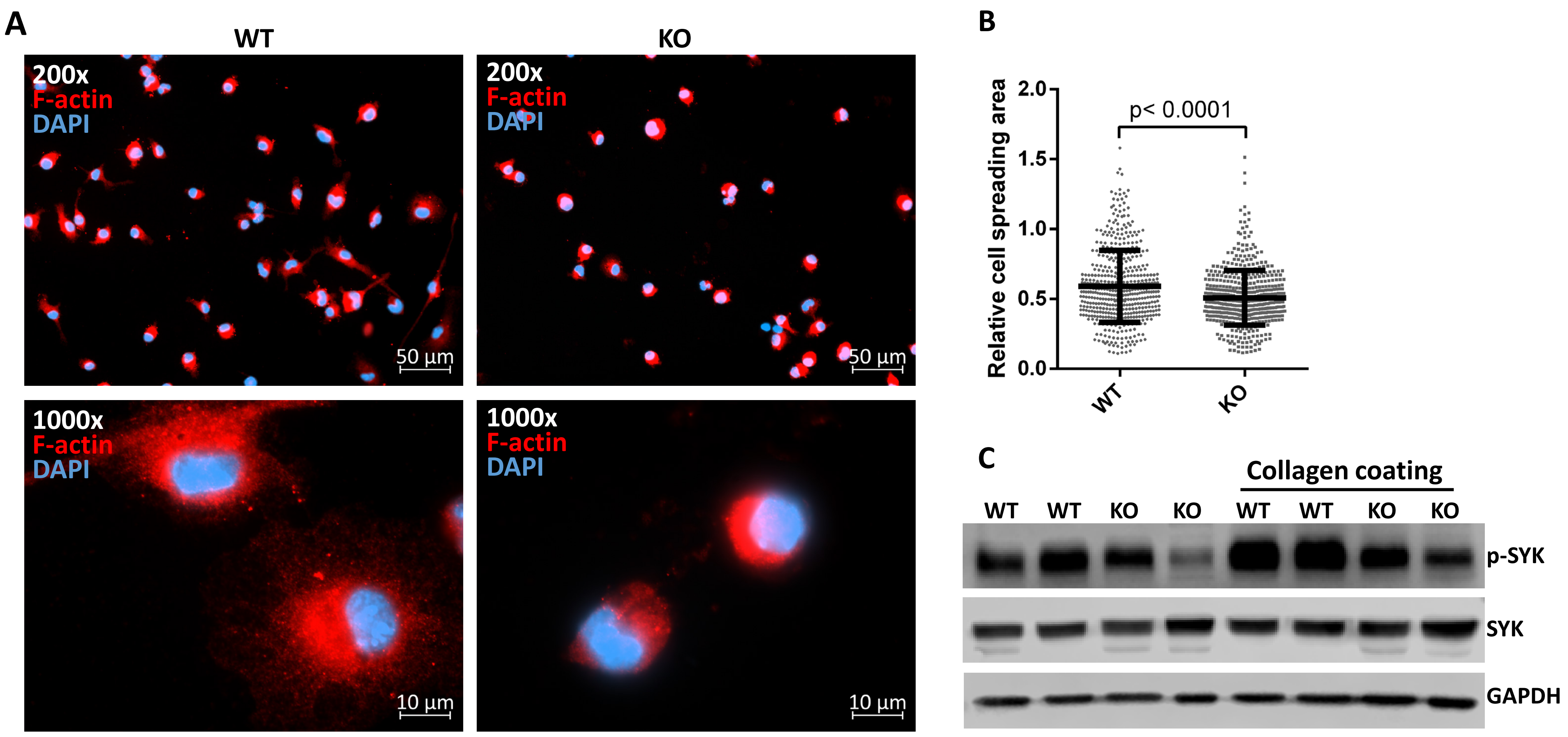

Figure S2. HCK KO in BMDMs impacted cell spreading by integrin signaling. Representative Immunofluorescence (IF) staining images (A) and quantification of cell spreading size (B) for F-actin in BMDM from WT and HCK KO mice. (C) Western blots for phospho-SYK show HCK KO degreased integrin signaling in BMDM.

A

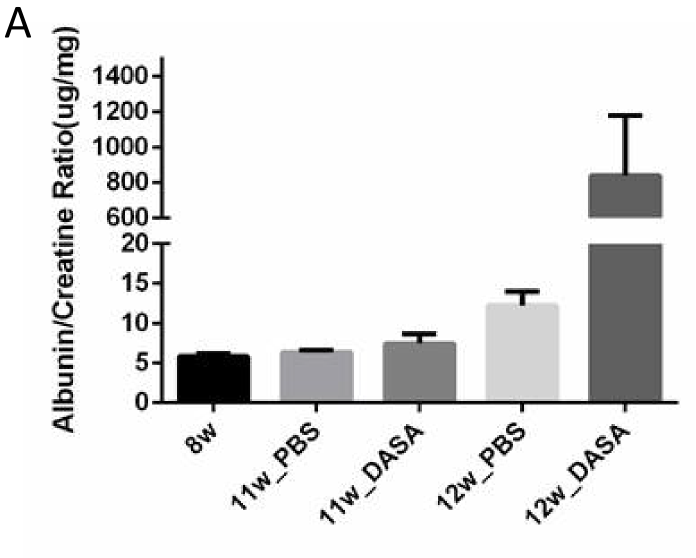

Figure S3. High dosage of dasatinib caused proteinuria in lupus mice. Urinary albumin-to-creatinine ratio was measured for lupus mice from 8 to 12 weeks with high dose of dasatinib treatment at 20mg/kg. DASA: dasatinib.

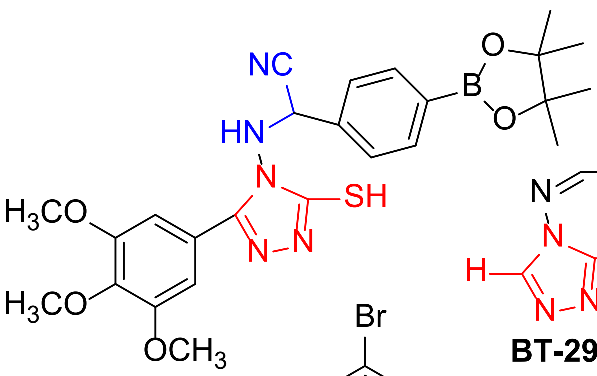

BT-291

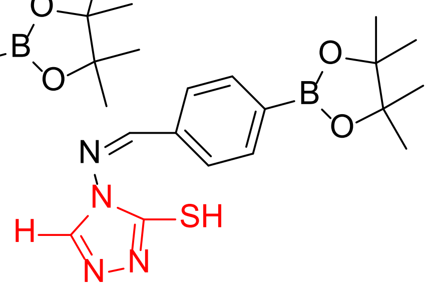

BT-292

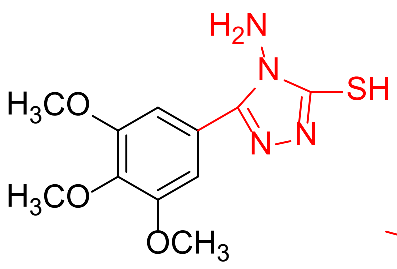

BT-296

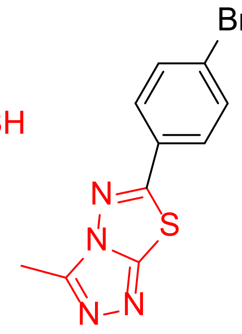

BT-293

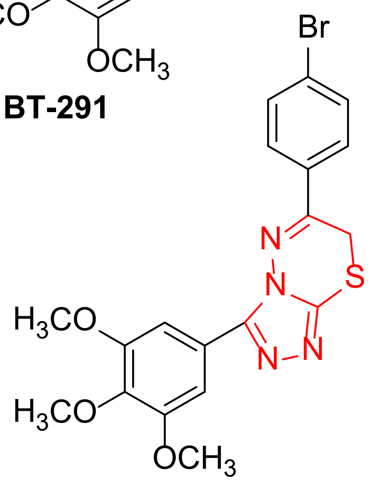

BT-294- Active

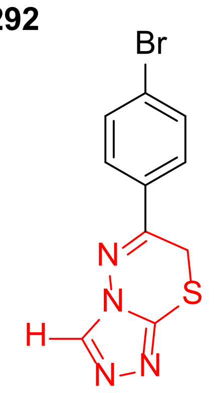

BT-295

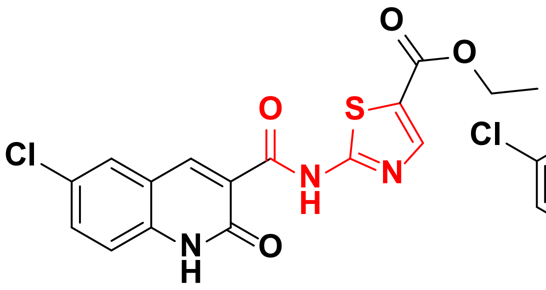

BT-331

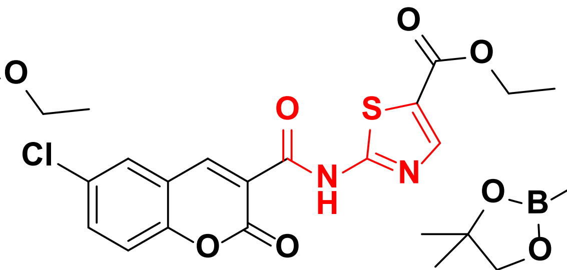

BT-332

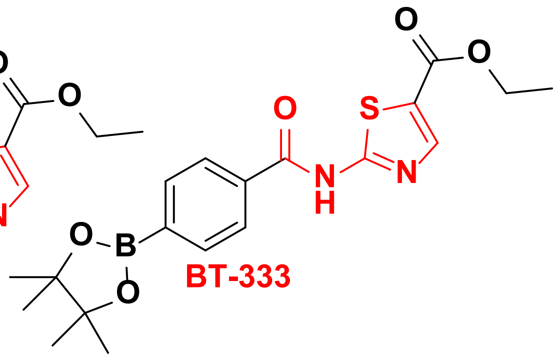

BT-333

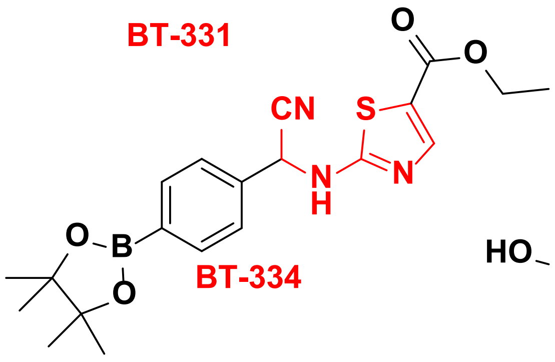

BT-334

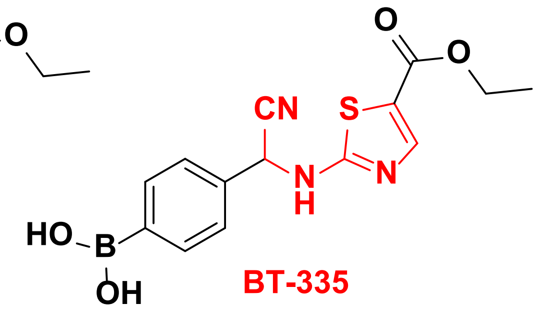

BT-335

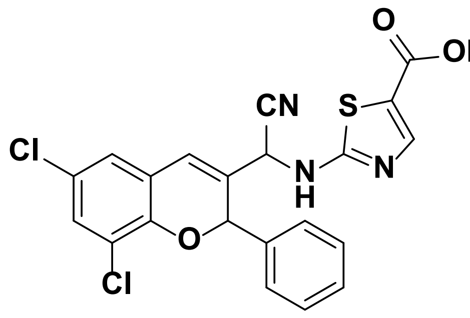

BT-339
MW: 485.0368

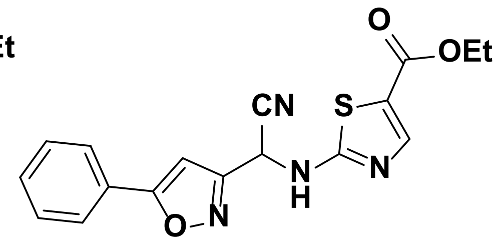

BT-340
MW: 354.0787

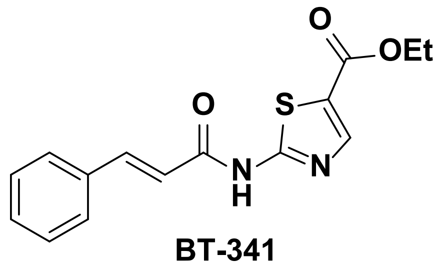

BT-341
MW:302.0725

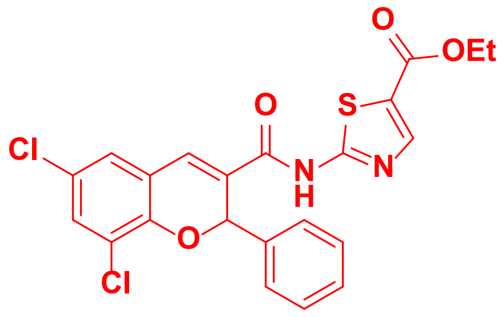

BT-342
MW:474.0208

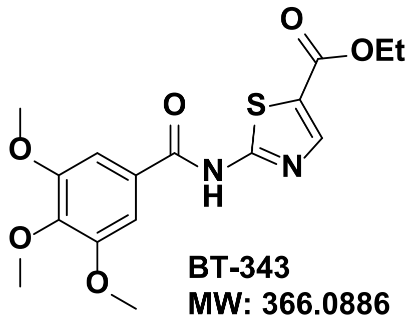

BT-343
MW: 366.0886

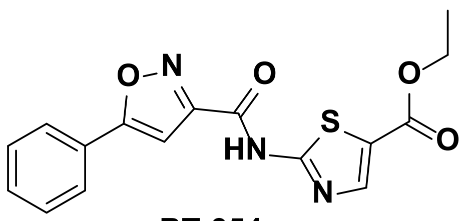

BT-354
MW:343.0627

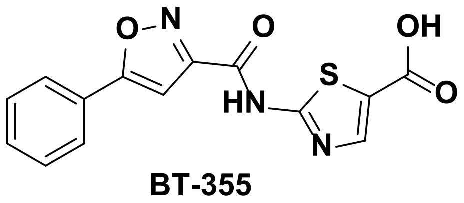

BT-355
MW: 315.0314

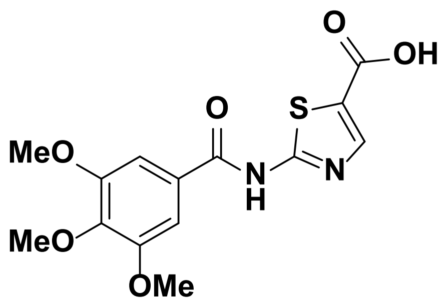

BT-356
MW:338.0573

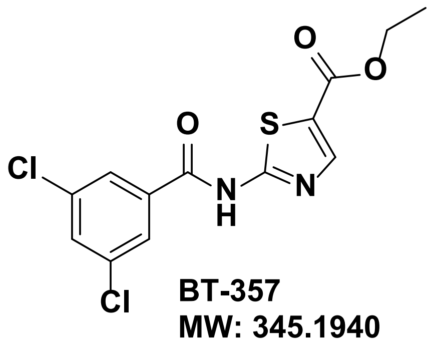

BT-357
MW: 345.1940

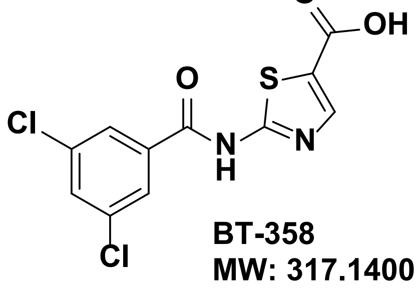

BT-358
MW: 317.1400

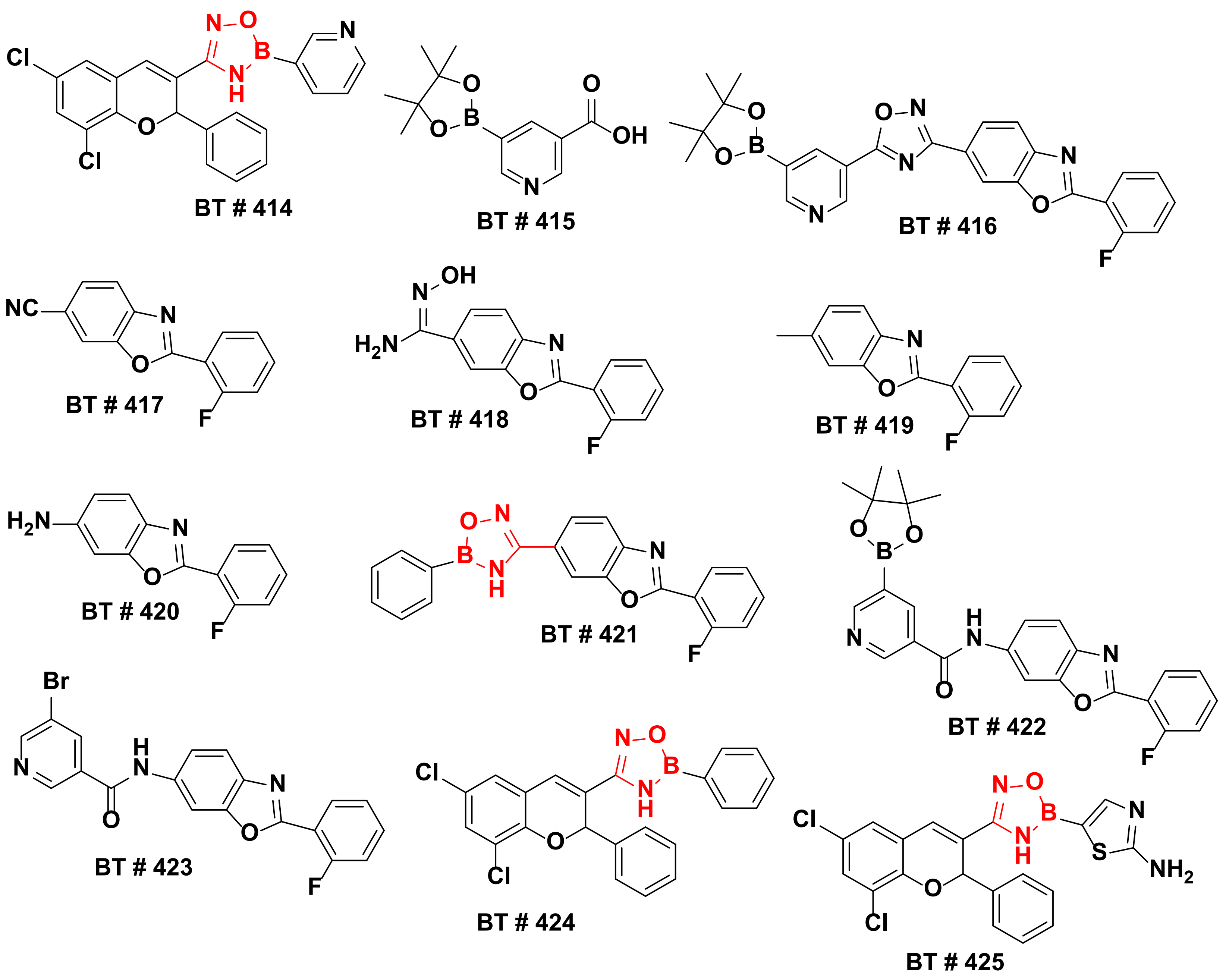

Figure S4. Chemical strictures of 33 designed and synthesized compounds HCK inhibitors.

A

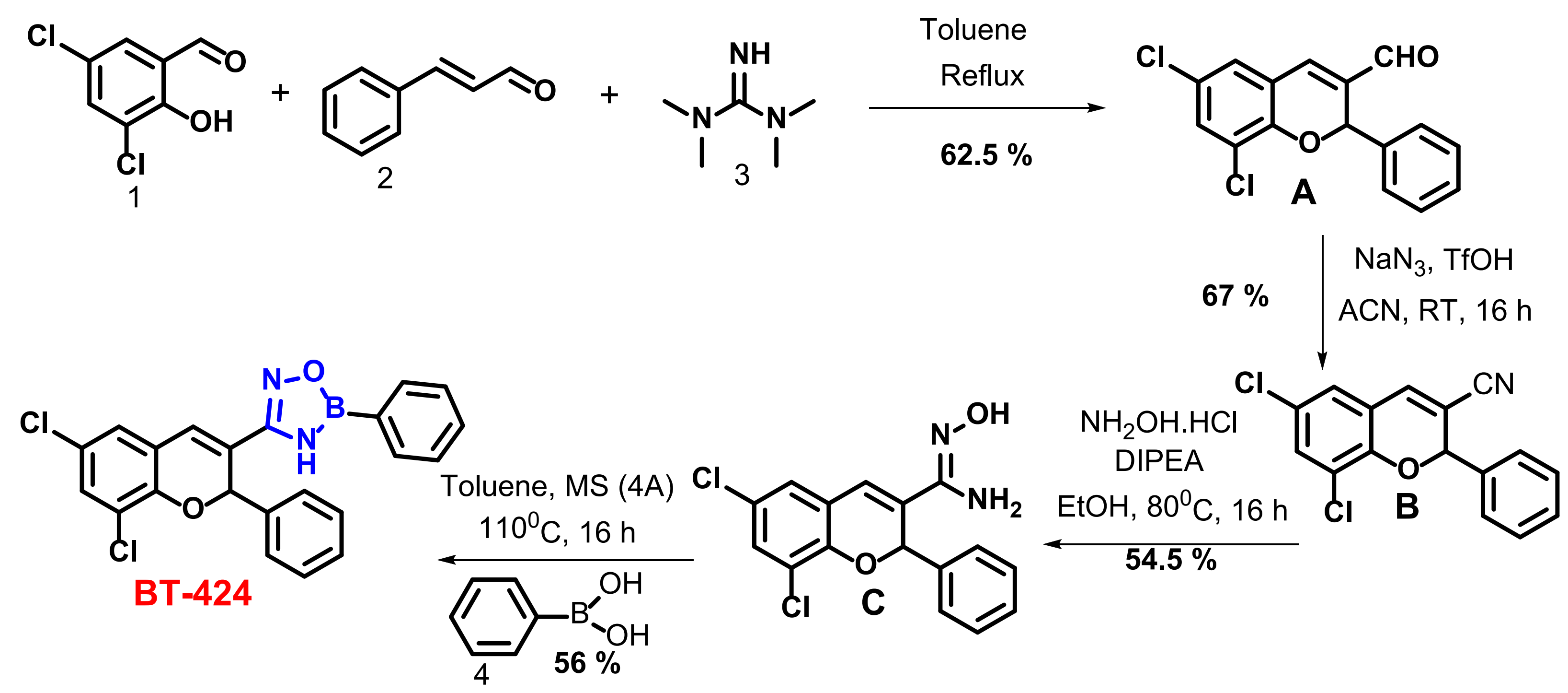
Cl
OH
Cl
1
2
NH
N
N
3
Toluene
Reflux
62.5 %
Cl
CHO
O
Cl
A
NaN3, TfOH
ACN, RT, 16 h
67 %
Cl
CN
O
Cl
B
NH2OH.HCl
DIPEA
EtOH, 80⁰C, 16 h
54.5 %
N OH
NH2
Cl
C
Toluene, MS (4A)
110⁰C, 16 h
OH
B
OH
4
56 %
N-O
B
N
H
BT-424

B

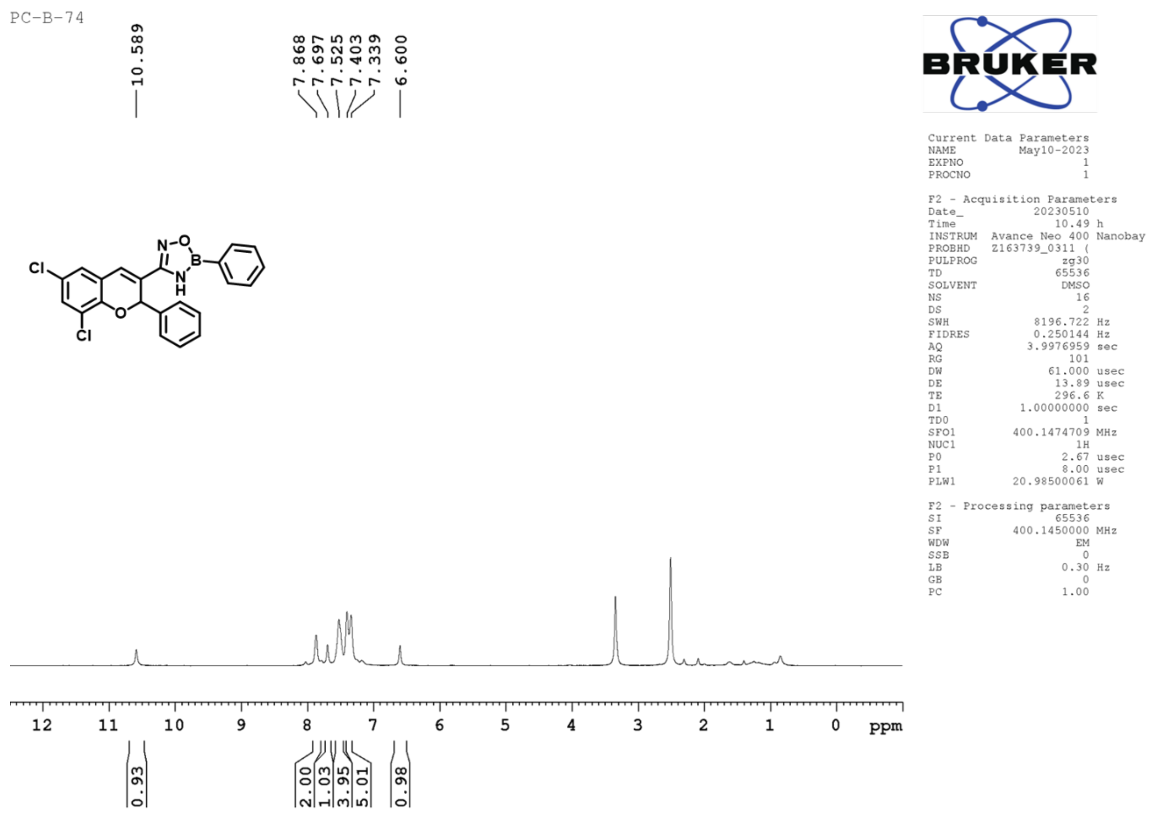
PC-B-74
10.589
7.868
7.697
7.525
7.403
7.339
6.600
BRUKER
12
11
10
9
8
7
6
5
4
3
2
1
0
ppm
0.93
2.00
1.03
3.95
5.01
0.98

C

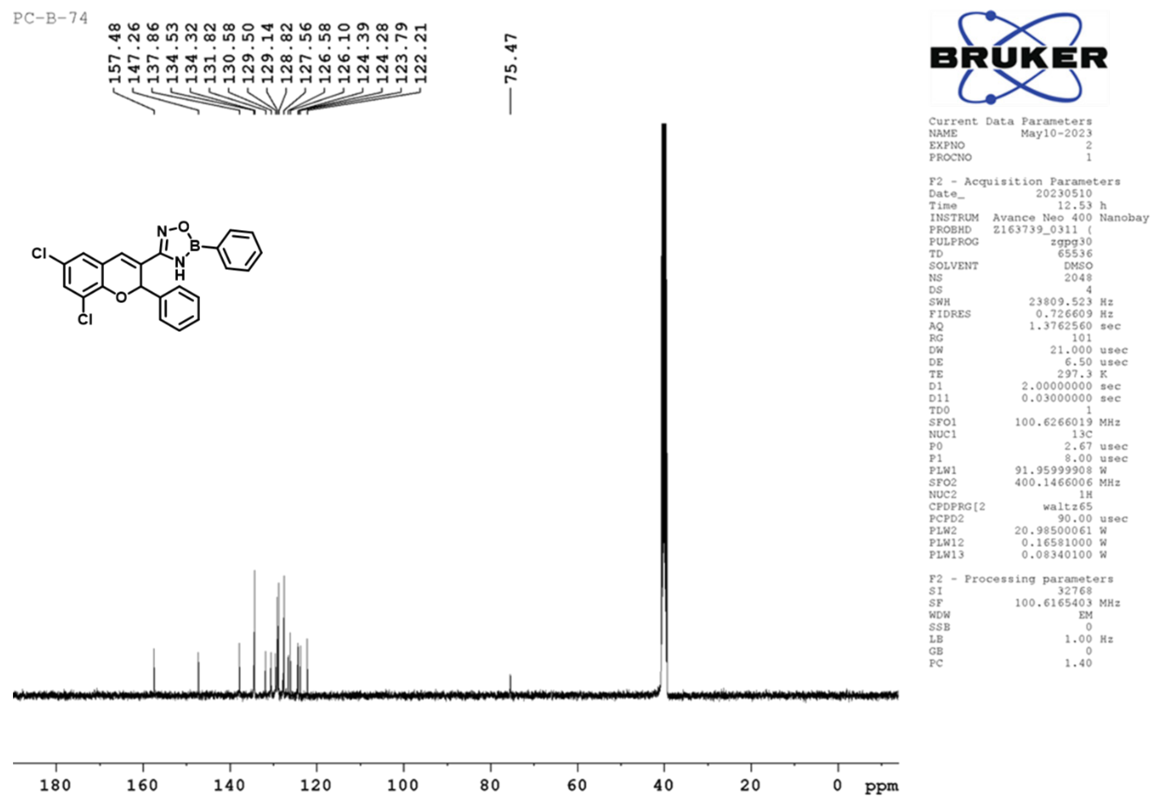
PC-B-74
157.48
147.26
137.86
134.53
134.32
131.82
130.58
129.50
129.14
128.82
127.56
126.58
126.10
124.39
124.28
123.79
122.21
75.47
BRUKER
180
160
140
120
100
80
60
40
20
0
ppm

D

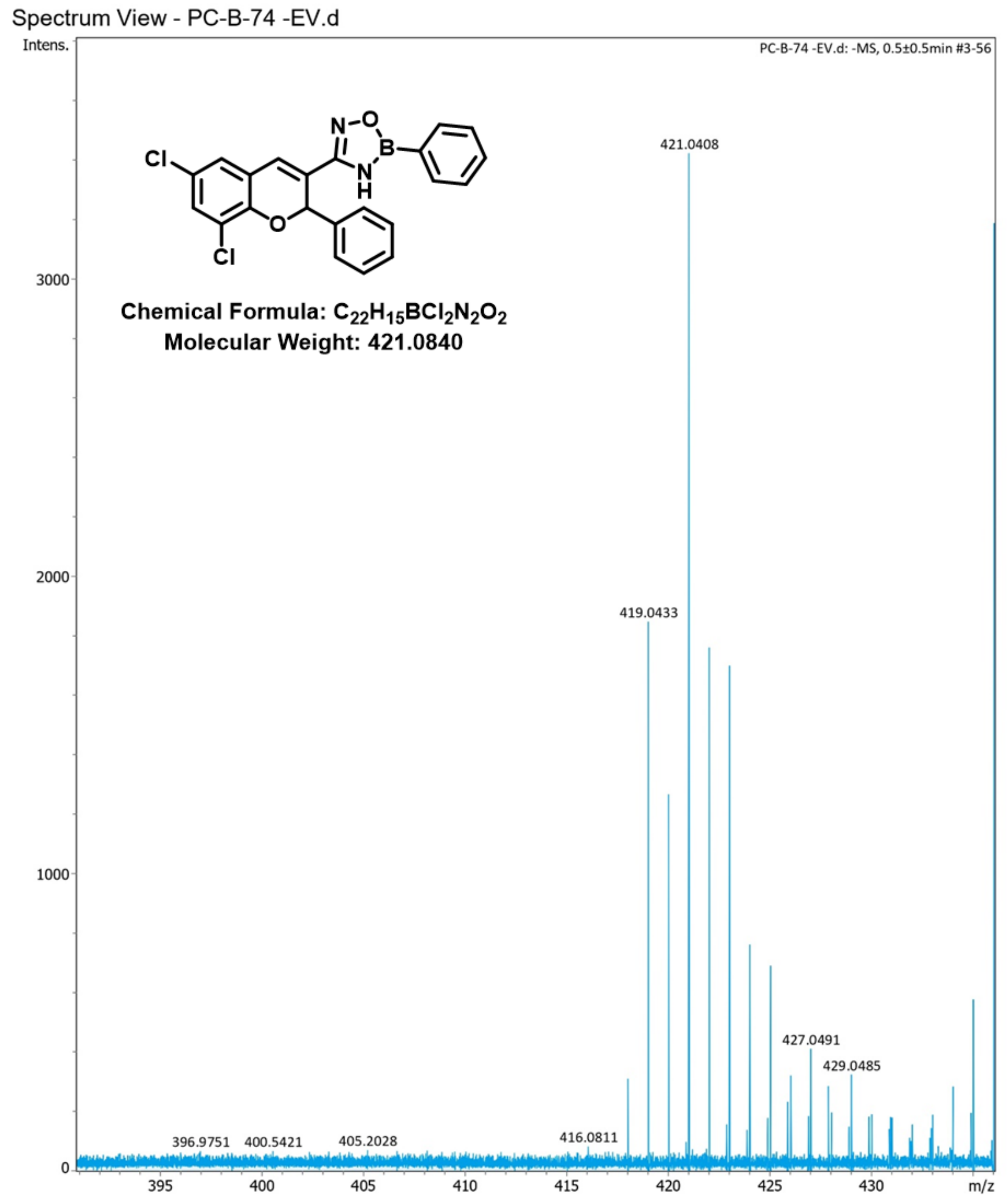
Spectrum View - PC-B-74 -EV.d
Intens.
PC-B-74 -EV.d: -MS, 0.5±0.5min #3-56
Chemical Formula: C22H15BCl2N2O2
Molecular Weight: 421.0840
421.0408
419.0433
427.0491
429.0485
396.9751
400.5421
405.2028
416.0811
3000
2000
1000
0
395
400
405
410
415
420
425
430
m/z

Figure S5. Synthesis procedure of 3-(6,8-dichloro-2-phenyl-2*H*-chromen-3-yl)-5-phenyl-4,5-dihydro-1,2,4,5-oxadiazaborole (BT-424).

(A) Step-by-step experimental procedure for BT-424 synthesis.

Procedure for the synthesis of 6,8-dichloro-2-phenyl-2H-chromene-3-carbonitrile B): A clean oven dried 20 mL round bottom flask was charged with 6,8-dichloro-2-phenyl-2*H*-chromene-3-carbaldehyde (**1)** (0.3 mmol), sodium azide (**2)** (0.4 mmol), and triflic acid (0.9 mmol), in acetonitrile (2 mL). The reaction mixture was stirred at room temperature for 16 h. Reaction progress was monitored by TLC. After completion of the reaction, the reaction mass was allowed to cool at ambient temperature, then acetonitrile was evaporated under reduced pressure, diluted with water, and extracted with EtOAc. The combined organic layer was dried with anhydrous Na_2SO_4 and evaporated under reduced pressure. The crude material was purified by column chromatography. The compound was obtained as a brown solid (1 g, yield 67 %).

^{1}H NMR (400 MHz, DMSO-d_6) δ 7.79 (s, 1H), 7.65 (s, 1H), 7.46 (s, 5H), 6.43 (s, 1H)

Procedure for the synthesis of (Z)-6,8-dichloro-N'-hydroxy-2-phenyl-2H-chromene-3-carboximidamide C): A clean oven dried 20 mL round bottom flask was charged with **1** (0.6 mmol), hydroxylammonium chloride (**2**) (1.3 mmol), and DIPEA (1.3 mmol), in ethanol (2 mL). The resulting reaction mixture was refluxed for 12 h. Reaction progress was monitored by TLC. After completion of the reaction, the ethanol was evaporated and extracted with EtOAc. The crude material was purified by column chromatography. The compound was obtained as a pale yellow solid (600 mg, yield 54.5 %).

^{1}H NMR (400 MHz, DMSO-d_6) δ 10.13 (s, 1H), 7.38 (t, *J* = 2.0 Hz, 1H), 7.31 (d, *J* = 4.1 Hz, 5H), 7.21 (t, *J* = 2.0 Hz, 1H), 6.44 (s, 1H), 5.81 (s, 2H); ^{13}C NMR (101 MHz, DMSO-d_6) δ 149.27, 147.05, 138.59, 129.76, 129.15, 128.99, 128.82, 127.59, 125.74, 125.70, 125.55, 121.93, 120.19, 75.01.

Procedure for the synthesis of 3-(6,8-dichloro-2-phenyl-2H-chromen-3-yl)-5-phenyl-4,5-dihydro-1,2,4,5-oxadiazaborole (BT-424):

A clean oven dried 10 mL round bottom flask was charged with **C** (0.5 mmol), phenylboronic acid (**4**) (0.6 mmol), and dissolved in toluene (3 mL). The reaction mixture was refluxed for 16 h in the presence of MS (4A). Reaction progress was monitored by TLC. After completion of the reaction, toluene was evaporated and the reaction mixture was diluted with acetone. After extracting with acetone and filtration, the filtrate was concentrated under reduced pressure. The residue was crystallized by an ethyl acetate-petroleum ether (1:4) mixture to obtain a pure product. The compound was obtained as a pale brown solid (70 mg, yield 56 %).

^{1}H NMR (400 MHz, DMSO-d_6) δ 10.59 (s, 1H), 7.87 (s, 2H), 7.70 (s, 1H), 7.53 (s, 3H), 7.37 (d, *J* = 25.5 Hz, 5H), 6.60 (s, 1H); ^{13}C NMR (101 MHz, DMSO-d_6) δ 157.50, 147.28, 137.88, 134.55, 134.34, 131.84, 130.60, 129.52, 129.16, 128.83, 127.58, 126.60, 126.11, 124.41, 124.30, 123.81, 122.23, 75.48; MS: calcd. for $C_{22}H_{15}BCl_2N_2O_2$ H^- 421.0840, found 421.0408.

(B) Proton nuclear magnetic resonance (NMR) spectrum, (C) carbon NMR spectrum, and (D) high resolution mass spectrometry (HRMS) spectrum for BT-424.

Supplementary Table S1. Serum chemistry test for mice treated with HCK selected inhibitor BT424 and vehicle.

	PBS-1	PBS-2	PBS-3	PBS-4	BT424-1	BT424-2	BT424-3	BT424-4
ALP(U/L)	74	65	106	97	72	61	73	--
AST(U/L)	47	--	63	41	33	30	28	48
ALT(U/L)	15	22	28	27	23	16	14	15
Creatine kinase(U/L)	32	--	108	67	39	23	26	--
GGT(U/L)	0	--	0	0	0	0	0	0
Albumin(g/dL)	3.1	2.5	2.4	3.1	2.9	2.7	2.9	--
Total Bilirubin(mg/dL)	0.2	--	0.1	0.2	0.1	0.1	0.1	--
Total Protein(g/dL)	--	--	4.3	5.3	5.1	4.8	4.8	--
Globulin(g/dL)	--	--	1.9	2.2	2.2	2.1	1.9	--
Bilirubin-Conjugated(mg/dL)	0	--	0	0	0	0	0	--
BUN (mg/dL)	32	--	29	31	24	27	22	28
Creatinine(mg/dL)	0	--	0	0	0	0	0	--
Cholesterol(mg/dL)	95	--	53	81	78	78	67	--
Glucose(mg/dL)	109	--	192	230	223	160	236	--
Calcium(mg/dL)	9.3	--	7.5	9.2	9.1	8.4	8.5	--
Phosphorus(mg/dL)	7.3	--	7.2	8.7	7.3	5.9	8.3	--
Bicarbonate TCO2(mmol/L)	13	--	16	15	17	12	12	--
ALB/GLOB ratio	--	--	1.3	1.4	1.3	1.3	1.5	--
BUN/Creatinine Ratio	0	--	0.1	0.2	0.1	0	0	--
Bilirubin - Unconjugated(mg/dL)	0.2	--	0.1	0.2	0.1	0.1	0.1	--
Magnesium(mg/dL)	3.2	--	2.8	2.9	3.4	2.5	2.9	--
Triglycerides (mg/dL)	76	--	81	81	110	107	82	--
Hemolysis Index	Normal	Normal	Normal	Normal	Normal	Normal	Normal	Normal
Lipemia Index	Normal	Normal	Normal	Normal	Normal	Normal	Normal	Normal

HEMOLYSIS

Normal, + Exhibits no significant effect on chemistry values.

++ Exhibits no significant effect on chemistry values.

+++ May increase AST by 25-50% and decrease ALP and Direct Bilirubin by 25-50%.

++++ May increase AST and CPK by 25 -50%, decrease ALP by > 50%, decrease Total and Direct Bilirubin by 25-50%, and decrease SDMA by 10-25%.

LIPEMIA

Normal, +, ++ Exhibits no significant effect on chemistry values.

+++ May decrease Direct Bilirubin by 25-50%.

++++ May decrease ALT, AST and Direct Bilirubin values by >50%.
