# Supplementary material for: HCK induces macrophage activation to promote renal inflammation and fibrosis via suppression of autophagy
Source: Nat Commun. 2023 Jul 18;14:4297. doi: 10.1038/s41467-023-40086-3 (PMC10354075; doi:10.1038/s41467-023-40086-3)
Supplement: Supplementary file 3 — Description of Additional Supplementary Files [file 41467_2023_40086_MOESM3_ESM.pdf]

Description of Additional Supplementary Files

Supplementary Movies 1-12: 3D-migration of BMDMs in Matrigel® Matrix with long-time-livecell imaging for groups of WT (V1-V4), HCK KO (V5-V8) and HCK inhibitor treated (V9-V12). WT, HCK KO and dasatinib pretreated BMDMs were seeded in Matrigel® Matrix in ibidi 8 well high glass bottom chamber. All BMDMs were recorded by long-time-live-cell microscopy for every 3 mins for 10 hours. Migrating cells were tracked from projected stacks to analyze velocity and persistence over time with ImageJ software TrackMate plugins. Each circle indicated one cell and the cell move track were indicated.
