# Supplementary material for: HCK induces macrophage activation to promote renal inflammation and fibrosis via suppression of autophagy
Source: Nat Commun. 2023 Jul 18;14:4297. doi: 10.1038/s41467-023-40086-3 (PMC10354075; doi:10.1038/s41467-023-40086-3)
Supplement: Supplementary file 16 — Reporting Summary [file 41467_2023_40086_MOESM16_ESM.pdf]

nature portfolio

Corresponding author(s): Bhaskar Das, John Cijiang He, Chengguo Wei

Last updated by author(s): Jun 23, 2023

Reporting Summary

Nature Portfolio wishes to improve the reproducibility of the work that we publish. This form provides structure for consistency and transparency in reporting. For further information on Nature Portfolio policies, see our Editorial Policies and the Editorial Policy Checklist.

Statistics

For all statistical analyses, confirm that the following items are present in the figure legend, table legend, main text, or Methods section.

n/a	Confirmed	
☐	☒	The exact sample size (*n*) for each experimental group/condition, given as a discrete number and unit of measurement
☐	☒	A statement on whether measurements were taken from distinct samples or whether the same sample was measured repeatedly
☐	☒	The statistical test(s) used AND whether they are one- or two-sided<br>*Only common tests should be described solely by name; describe more complex techniques in the Methods section.*
☒	☐	A description of all covariates tested
☒	☐	A description of any assumptions or corrections, such as tests of normality and adjustment for multiple comparisons
☐	☒	A full description of the statistical parameters including central tendency (e.g. means) or other basic estimates (e.g. regression coefficient) AND variation (e.g. standard deviation) or associated estimates of uncertainty (e.g. confidence intervals)
☐	☒	For null hypothesis testing, the test statistic (e.g. *F*, *t*, *r*) with confidence intervals, effect sizes, degrees of freedom and *P* value noted<br>*Give P values as exact values whenever suitable.*
☒	☐	For Bayesian analysis, information on the choice of priors and Markov chain Monte Carlo settings
☒	☐	For hierarchical and complex designs, identification of the appropriate level for tests and full reporting of outcomes
☒	☐	Estimates of effect sizes (e.g. Cohen's *d*, Pearson's *r*), indicating how they were calculated

Our web collection on statistics for biologists contains articles on many of the points above.

Software and code

Policy information about availability of computer code

Data collection	Attune Flow Cytometry for PI stain<br>AxioImager. Z2(M) for IHC IF microscopy image<br>Image Studio Lite Ver5.2 for western blot image<br>Leica DMIRBE microscope for live cell migration imaging.
Data analysis	R x64 4.03 for HCK expression micro-array data analysis - https://www.r-project.org/<br>Image J 1.52a for stain microscopy image analysis - https://imagej.net/ij/index.html<br>ZEN 2.3 for AxioImager. Z2(M) (Motorized) - https://www.zeiss.com/microscopy/en/products/software/zeiss-zen-lite.html<br>FlowJo v10.8.0 for flow cytometry data analysis - https://www.flowjo.com/<br>Image Studio Lite Ver5.2 for western blot image analysis - https://www.licor.com/bio/image-studio-lite/<br>LAS X 3.7.4 for live cell imaging of cell migration - https://www.leica-microsystems.com/

For manuscripts utilizing custom algorithms or software that are central to the research but not yet described in published literature, software must be made available to editors and reviewers. We strongly encourage code deposition in a community repository (e.g. GitHub). See the Nature Portfolio guidelines for submitting code & software for further information.

Data

Policy information about availability of data

All manuscripts must include a data availability statement. This statement should provide the following information, where applicable:

- Accession codes, unique identifiers, or web links for publicly available datasets
- A description of any restrictions on data availability
- For clinical datasets or third party data, please ensure that the statement adheres to our policy

The high-resolution video files of cell 3D-random migration are too large to upload as supplementary files and are available from the corresponding authors on reasonable request. All other relevant data supporting the key findings of this study are available within the article and its Supplementary Information files. Source data are provided with this paper.

Research involving human participants, their data, or biological material

Policy information about studies with human participants or human data. See also policy information about sex, gender (identity/presentation), and sexual orientation and race, ethnicity and racism.

Reporting on sex and gender	Both male and female were included in the patient kidney biopsy collection. Please find more information on our previous paper PMID: 27452608 Lancet 2016; 388: 983–93.
Reporting on race, ethnicity, or other socially relevant groupings	All the race were included in the patient kidney biopsy collection. Please find more information on our previous paper PMID: 27452608 Lancet 2016; 388: 983–93.
Population characteristics	Please find more information on our previous paper PMID: 27452608 Lancet 2016; 388: 983–93.
Recruitment	Please find more information on our previous paper PMID: 27452608 Lancet 2016; 388: 983–93.
Ethics oversight	The clinical data from the 5 clinical centers (Icahn school of Medicine at Mount Sinai, New York; Westmead clinical school, University of Sydney, Sydney; Northwestern University Feinberg School of Medicine, Chicago; University of Michigan, Ann Arbor; University of Wisconsin, Madison), the central pathology core (Massachussets general hospital, Harvard Medical School, Boston) and immunological core (Brigham and Women's Hospital, Harvard Medical School, Boston) were deposited into a central clinical eRAP database developed at Mount Sinai.<br>Informed written consent was obtained from all study participants from the individual clinical sites at the time of enrollment into the original study protocol. IRB approval was obtained from all participating institutions.

Note that full information on the approval of the study protocol must also be provided in the manuscript.

Field-specific reporting

Please select the one below that is the best fit for your research. If you are not sure, read the appropriate sections before making your selection.

[x] Life sciences [ ] Behavioural & social sciences [ ] Ecological, evolutionary & environmental sciences

For a reference copy of the document with all sections, see nature.com/documents/nr-reporting-summary-flat.pdf

Life sciences study design

All studies must disclose on these points even when the disclosure is negative.

Sample size	No power analysis was performed to determine the sample size. For mice experiments, We used 8 mice (n=8) in each group. In vitro studies were performed with a minimum 3 (n=3) biological replication.
Data exclusions	No data was excluded.
Replication	All experiments were repeated three times with similar results. Only the cytokine array only conducted once as the high cost.
Randomization	For all the samples in mouse and cell experiments, they were allocated to different groups randomly.
Blinding	All experiments in our study were blinded to investigators during group allocation. All quantification analyses for mouse and in vitro studies were also blinded for investigators.

Reporting for specific materials, systems and methods

We require information from authors about some types of materials, experimental systems and methods used in many studies. Here, indicate whether each material, system or method listed is relevant to your study. If you are not sure if a list item applies to your research, read the appropriate section before selecting a response.

Materials & experimental systems

n/a	Involved in the study
	☒ Antibodies
	☒ Eukaryotic cell lines
☒	☐ Palaeontology and archaeology
	☒ Animals and other organisms
	☒ Clinical data
☒	☐ Dual use research of concern
☒	☐ Plants

Methods

n/a	Involved in the study
☒	☐ ChIP-seq
	☒ Flow cytometry
☒	☐ MRI-based neuroimaging

Antibodies

Antibodies used	(1) Western Blotting:<br>Hck (E1I7F) Rabbit mAb (CST #14643), V5 tag (GenScript Inc #A01724), phosphor-Y410 HCK (Abcam #ab61055), LC3A/B (CST #4108), Fyn (CST #4023S), p62/SQSTM1 (NOVUS BIOLOGICALS NBP1-48320SS), Atg2A (CST #15011), c-Cbl (CST #2747), phospho-c-Cbl (Tyr700) (D16D7) (CST #8869), MMR/CD206 (R&D Systems #AF2535), iNOS (BioLegend #690902), Arginase 1 (BioLegend #678802), Phospho-PI3K p85 /p55 (CST #17366), Phospho-Akt (CST #4060), Akt (CST #4691), Phospho-p44/42 MAPK (Erk1/2) (CST #9101), p44/42 MAPK (Erk1/2) (CST #9102), EGFR (CST #4267), Phospho-EGFR (CST #3777), SYK (CST #13198), pY20 (Invitrogen # 14-5001-82), a-SMA (Sigma-Aldrich #A5228) and GAPDH mAb (CST #2118). Anti-V5-tag mAb-Magnetic Beads (MBL #M167-11) were used for immunoprecipitation.<br>Antibody anti-GAPDH (14C10) was diluted 5000 times with 3% BSA in PBST. All other antibodies mentioned above for WB were used dilution of 1:1000. The Anti-V5-tag mAb-Magnetic Beads were used 50ul for each reaction with 400ul incubation volume. The secondary antibodies were from Promega: Anti-Rabbit IgG (H+L), HRP Conjugate (#W4011) and Anti-Mouse IgG (H+L), HRP Conjugate (#W4021). The secondary antibodies were diluted with 1:5000.<br>(2) Immunofluorescence staining:<br>Anti-phospho Y410 HCK antibody (Abcam, #ab61055), anti-HCK antibody (Abcam, ab75839 or Cell Signaling Technology, #14643), anti-LC3A/B antibody (Cell Signaling Technology, #4108), anti-Ki67 antibody (Abcam, ab16667), anti-F4/80 antibody (Invitrogen, 14-4801-82), anti-COL1A1 antibody (Southern Biotech, #1310-01), anti-F-actin antibody (Novus Biologicals, #NB100-64792).<br>All antibodies mentioned above for IF staining were used dilution of 1:100. The secondary antibodies were from Invitrogen: Goat anti-Rat IgG (H+L) Cross-Adsorbed Secondary Antibody, Alexa Fluor™ 568, Goat anti-Rabbit IgG (H+L) Cross-Adsorbed Secondary Antibody, Alexa Fluor™ 568, Goat anti-Rabbit IgG (H+L) Highly Cross-Adsorbed Secondary Antibody, Alexa Fluor™ 488, Goat anti-Mouse IgG (H+L) Cross-Adsorbed Secondary Antibody, Alexa Fluor™ 488, Goat anti-Mouse IgG (H+L) Cross-Adsorbed Secondary Antibody, Alexa Fluor™ 568. The secondary antibodies were diluted with 1:200.
Validation	All antibodies used in this study were commercial and validated by the manufacturer. Species and application validations and citations for primary antibodies can be found from the manufacturer's websites.

Eukaryotic cell lines

Policy information about cell lines and Sex and Gender in Research

Cell line source(s)	RAW264.7 (TIB-71™), HEK293 (CRL-1573™) and NCTC clone 929 (CCL-1™) cells commercially obtained from ATCC. BMDM were isolated from mice.
Authentication	All cells were authenticated by short tandem repeat (STR) analysis.
Mycoplasma contamination	All lines tested negative for mycoplasma using the Sigma Look-Out PCR Detection Kit.
Commonly misidentified lines<br>(See ICLAC register)	No commonly misidentified cell lines were used in the study.

Animals and other research organisms

Policy information about studies involving animals; ARRIVE guidelines recommended for reporting animal research, and Sex and Gender in Research

Laboratory animals	HCK exon3 loxp flanked 15 transgenic mouse at EuMMCR in Germany. C57BL/6J WT, CMV-Cre (#006054) and LysM-Cre (# 004781) mice were purchased from the Jackson Laboratory. All mice were maintained in our animal facility under controlled environmental conditions: 12/12 light/dark cycle, ambient temperature 20-25°C. More information can be found through their website: https://icahn.mssm.edu/research/ccms. Mice at 8-10 week age were used for experiments in this study.
Wild animals	No wild animals were used in the study.
Reporting on sex	Male and female were both included in each experiment.
Field-collected samples	No field collected samples were used in the study.

Ethics oversight	All animal studies were approved and overseen by the Institute for Animal Care and Use Committee at Icahn School of Medicine at Mount Sinai.

Note that full information on the approval of the study protocol must also be provided in the manuscript.

Clinical data

Policy information about clinical studies

All manuscripts should comply with the ICMJEguidelines for publication of clinical research and a completedCONSORT checklist must be included with all submissions.

Clinical trial registration	N/A
Study protocol	STUDY-11-01259
Data collection	Protocol biopsy were collect form patient kidney 12 month after transplantation. Macro-array were performed to obtain gene expression.
Outcomes	N/A

Flow Cytometry

Plots

Confirm that:

- [x] The axis labels state the marker and fluorochrome used (e.g. CD4-FITC).
- [x] The axis scales are clearly visible. Include numbers along axes only for bottom left plot of group (a 'group' is an analysis of identical markers).
- [x] All plots are contour plots with outliers or pseudocolor plots.
- [x] A numerical value for number of cells or percentage (with statistics) is provided.

Methodology

Sample preparation	RAW264.7 or BMDM were stained with propidium iodide (50ug/ml) for 15 mins.
Instrument	Attune Flow Cytometers
Software	Attune
Cell population abundance	10E6/ml
Gating strategy	Forward scatter (FS) and side scatter (SS) were used to identify single cells, and all these cells were used for cell cycle analysis with propidium iodide.

- [x] Tick this box to confirm that a figure exemplifying the gating strategy is provided in the Supplementary Information.
